# Shunt dependency in supratentorial intraventricular tumors depends on the extent of tumor resection

**DOI:** 10.1007/s00701-023-05532-7

**Published:** 2023-03-02

**Authors:** Nico Teske, Mariana Chiquillo-Domínguez, Benjamin Skrap, Patrick N. Harter, Kai Rejeski, Jens Blobner, Louisa von Baumgarten, Joerg-Christian Tonn, Mathias Kunz, Niklas Thon, Philipp Karschnia

**Affiliations:** 1 Department of Neurosurgery, University Hospital, LMU Munich, Marchioninistrasse 15, 81377 Munich, Germany; 2 German Cancer Consortium (DKTK), Partner Site Munich, Munich, Germany; 3 Center for Neuropathology and Prion Research, University Hospital, LMU Munich, Munich, Germany; 4 Department of Medicine III, University Hospital, Ludwig-Maximilians-University Munich, Munich, Germany

**Keywords:** Supratentorial intraventricular tumors, Hydrocephalus, Shunting, Extent of resection

## Abstract

**Background:**

Supratentorial intraventricular tumors (SIVTs) are rare lesions of various entities characteristically presenting with hydrocephalus and often posing a surgical challenge due to their deep-seated localization. We aimed to elaborate on shunt dependency after tumor resection, clinical characteristics, and perioperative morbidity.

**Methods:**

We retrospectively searched the institutional database for patients with supratentorial intraventricular tumors treated at the Department of Neurosurgery of the Ludwig-Maximilians-University in Munich, Germany, between 2014 and 2022.

**Results:**

We identified 59 patients with over 20 different SIVT entities, most often subependymoma (8/59 patients, 14%). Mean age at diagnosis was 41 ± 3 years. Hydrocephalus and visual symptoms were observed in 37/59 (63%) and 10/59 (17%) patients, respectively. Microsurgical tumor resection was provided in 46/59 patients (78%) with complete resection in 33/46 patients (72%). Persistent postoperative neurological deficits were encountered in 3/46 patients (7%) and generally mild in nature. Complete tumor resection was associated with less permanent shunting in comparison to incomplete tumor resection, irrespective of tumor histology (6% versus 31%, *p* = 0.025). Stereotactic biopsy was utilized in 13/59 patients (22%), including 5 patients who received synchronous internal shunt implantation for symptomatic hydrocephalus. Median overall survival was not reached and did not differ between patients with or without open resection.

**Conclusions:**

SIVT patients display a high risk of developing hydrocephalus and visual symptoms. Complete resection of SIVTs can often be achieved, preventing the need for long-term shunting. Stereotactic biopsy along with internal shunting represents an effective approach to establish diagnosis and ameliorate symptoms if resection cannot be safely performed. Due to the rather benign histology, the outcome appears excellent when adjuvant therapy is provided.

**Supplementary Information:**

The online version contains supplementary material available at 10.1007/s00701-023-05532-7.

## Introduction

Supratentorial intraventricular tumors (SIVTs) represent rare lesions accounting for approximately 1–3% of all intracranial lesions [16, 19]. They consist of a variety of tumor entities that either arise primarily from the ventricular system such as ependymal, glioneuronal, meningeal or choroid plexus tumors, or by metastatic intraventricular tumor growth from primary and secondary brain tumors with initial intraparenchymal growth [23, 27]. In comparison to infratentorial intraventricular tumors, SIVTs usually present with different symptoms, harbor unique perioperative risks, and necessitate distinct surgical approaches and shunting strategies [1, 35]. Patients most often present with symptoms consistent with obstructive hydrocephalus due to tumor mass effect on bottlenecks of cerebrospinal fluid (CSF) flow or with incidental imaging findings in the context of a diagnostic workup for unrelated symptoms. In general, first-line therapy for symptomatic SIVTs consists of complete microsurgical tumor resection or neuroendoscopic resection, followed by adjuvant therapy if necessary (depending on histological tumor entity). Exceptions are benign colloid or pineal cysts which can be managed with minimally invasive stereotactic procedures and pineal germ cell tumors which may be diagnosed by biopsy or CSF analyses prior to medical treatment [9].

From a surgical perspective, SIVTs pose a substantial challenge due to their deep-seated location, eloquent deep brain structures surrounding the walls of the supratentorial ventricular system, and critical neurovascular structures that have to be preserved during tumor resection [10]. In contrast to infratentorial intraventricular tumors, a multitude of surgical approaches exist that allow for maximal safe tumor resection while ensuring minimal brain retraction depending on tumor location within the ventricular system [6, 34]. In regards to alleviating obstructive hydrocephalus in the setting of SIVTs, a diverse set of shunting strategies exist including perioperative external ventricular drainage (EVD), ventriculoperitoneal (VP) or -atrial shunt placement, endoscopic third ventriculostomy, and stereotactic internal shunting (a minimally invasive stereotactic-guided catheter implantation technique that connects the supratentorial ventricular system with the basal cisterns and allow CSF drainage as previously described) [11, 15, 17, 35]. The preferred choice of shunting procedure and whether preoperative hydrocephalus necessitates shunting after complete tumor removal still remains unclear. In particular, stereotactic internal shunting is only offered at specialized centers and its effectivity in SIVTs is currently ill defined.

In the present study, we describe a large well-defined cohort of 59 patients who were consecutively treated for supratentorial intraventricular tumorous lesions at a single academic neuro-oncology center. Based on clinical and imaging findings, we herein focus on outlining different shunting strategies for patients with persistent hydrocephalus after surgery depending on the extent of resection, elaborate on surgical approaches and their outcomes, and discuss perioperative morbidity.

## Materials and methods

### Study population

We conducted a retrospective analysis of our institutional database within the framework of the Center for Neuro-Oncology at the Ludwig-Maximilians-University School of Medicine for patients presenting between 2014 and 2022 for SIVTs that were treated with either microsurgical tumor resection or stereotactic biopsy. Patients were selected based on the following criteria: (I) tumors at least partially located in the supratentorial ventricular system (i.e., lateral ventricles and third ventricle; excluding non-tumorous lesions such as colloid cysts); (II) tissue-based diagnosis according to the 2021 World Health Organization (WHO) classification of tumors of the central nervous system (CNS) [16]; (III) pre- and postoperative magnetic resonance imaging (MRI) available for review and volumetric data analyses (Supplementary Fig. 1). Data cutoff for the study cohort was March 1, 2022. Study protocol and design were approved by the Institutional Review Board of the Ludwig-Maximilians-University in Munich, Germany, and patient consent was waived (AZ 20–063). We collected demographic and clinical information, histopathology, imaging and other diagnostic findings, surgical and adjuvant treatment specifics, and outcome data. Postoperative deficits were defined as new or worsening neurological impairment including but not limited to seizures, cranial nerve deficits, aphasia, motor and sensory deficits, neurocognitive impairments, and a decreased level of consciousness. Non-life-threatening deficits not requiring surgical, endoscopic, or radiological intervention were considered mild to moderate in nature; in case of any intervention and/or necessary intensive care unit (ICU) management, deficits were classified as severe. Interdisciplinary brain tumor board recommendations and patient preference formed the basis for diagnostic and treatment decisions. Follow-up imaging and surveillance scans were obtained per institutional guidelines, depending on the respective tumor entities, or in case of any clinical deterioration.

### Magnetic resonance imaging

Magnetic resonance imaging at first diagnosis and at follow-up was evaluated according to the RANO criteria [33]. Tumor volumes were quantified by pre- and postoperative contrast-enhancing (CE) T1-weighted sequences and/or FLAIR (or if not available T2)-sequences (in case of non-CE lesions) using a commercially available imaging software (BrainLab® Elements; Munich). We ensured that postoperative FLAIR/T2-abnormalities did not reflect surgically induced edema or ischemia by reviewing T2- and diffusion-weighted imaging sequences. Absolute residual tumor volumes (in cm^3^) were recorded, and extent of resection (EOR, in percentage) was calculated. “Incomplete resection” of tumor was defined as ≥ 1 cm^3^ tumor volume and “complete resection” as < 1 cm^3^ tumor volume on postoperative MRI imaging, based on previously proposed tumor resection classifications [13]. Occlusive hydrocephalus was radiologically diagnosed in case of an (asymmetric) widening of the lateral ventricles and/or third ventricle, often accompanied by subsequent transependymal CSF diapedesis [14].

For quantitative apparent diffusion coefficient (ADC) analysis, regions of interest (ROIs) were manually placed within the solid tumor mass (avoiding cysts, hemorrhage, edema) and the lateral ventricles. ADC values were recorded as previously described [20, 22], using a dedicated image processing software (Visage 7; Visage Imaging, Inc., San Diego). Tumor ADC_mean_ values were normalized for CSF as follows: “tumor/CSF ratio = [ADC_mean_(tumor)/ADC_mean_(CSF)].”

### Neuropathological diagnosis

Histopathologic diagnosis was based on the 2021 WHO classification of CNS tumors from tissue sampled during microsurgical tumor resection or stereotactic biopsy [16].

O6-Methylguanine-DNA-methlytransferase (MGMT) promotor, telomerase reverse transcriptase promotor mutation, isocitrate dehydrogenase 1/2 and BRAF mutation status, as well as loss of heterozygosity of 1p/19q were analyzed as previously described [3, 28]. For extended molecular diagnostics, next-generation sequencing and DNA methylation-based tumor diagnostics were performed whenever deemed clinically necessary [4]. In case histologic grading criteria and molecular analyses could not yield sufficient information for definite tumor grading for exceedingly rare pineal parenchymal tumors of intermediate differentiation (PPTID) WHO CNS grade 2 or 3, tumors were allocated to grade 3 for statistical analyses [16, 21].

### Statistical analysis

Associations between two or more categorical variables were analyzed using the chi-squared test. Categorical variables were expressed in absolute numbers and percentages. The D’Agostino-Pearson omnibus normality test was used to test for normal distribution and equal variance in continuous data. The unpaired Student’s *t* test and ANOVA test were used to determine differences among two or more groups. In case of non-parametric data, Mann–Whitney *U*-test and the Kruskal–Wallis-test were used, respectively. Numerical data were described as mean ± standard error of the mean, and range was provided, if not indicated otherwise. For survival analyses, Kaplan–Meier survival estimates and log-rank tests were calculated. Patients were followed until data cutoff (March 1, 2022), or death. Individuals with a follow-up time of less than 1 month were excluded for survival analyses. Patients lost to follow-up were censored at day of last follow-up. Date of diagnosis was set as the date of biopsy or surgical tumor resection. Overall survival was defined as the interval from diagnosis to death from any cause, and progression-free survival was defined as the interval from diagnosis to progression or death from any cause. Radiographic progression was defined according to the RANO criteria [33]. The significance level was set at *p* ≤ 0.05. Statistical analyses were performed using Prism statistical software (Prism 9.4; GraphPad Software Inc., San Diego, CA, USA).

## Results

### Baseline patient characteristics and imaging findings

From a total of 1536 brain tumor patients treated at our Center for Neuro-Oncology between 2014 and 2022, 59 patients fulfilled the inclusion criteria (Supplementary Fig. 1). In these patients, the mean age at diagnosis was 41 ± 3 years (range: 0–86 years) with a male-to-female ratio of 1:0.9. Median preoperative Karnofsky performance status (KPS) was 90% (range: 20–100%, including two patients with acute hydrocephalus and decreased level of consciousness at presentation). At time of diagnosis, patients most often presented with symptoms suggestive of hydrocephalus (e.g., headache, nausea, emesis; 37/59 patients, 63%). However, visual symptoms (e.g., visual impairment, field deficits, gaze palsies) were also frequently encountered (10/59 patients, 17%). In contrast, tumors diagnosed on the basis of incidental imaging findings were observed in 9/59 patients (15%) (Table 1).

**Table 1:** Patient characteristics for supratentorial intraventricular tumors

		Biopsy	Incomplete resection	Complete resection	Total	*p*-value
Overall, *n* (%)		13 (22)	13 (22)	33 (56)	59 (100)	
Age, years	Mean	55.5	37.2	37.5	41.4	0.052
	< 18	2 (15)	1 (8)	7 (21)	10 (17)	
	18–35	2 (15)	6 (46)	10 (30)	18 (31)	
	36–50	0 (0)	2 (15)	4 (12)	6 (10)	
	51–65	3 (23)	2 (15)	8 (24)	13 (22)	
	> 65	6 (46)	2 (15)	4 (12)	12 (20)	
Sex	Female	4 (31)	5 (38)	19 (58)	28 (47)	0.199
	Male	9 (69)	8 (62)	14 (42)	31 (53)	
Clinical presentation						
Preoperative KPS, %	< 90	5 (38)	4 (31)	4 (12)	13 (22)	0.412
	90–100	8 (62)	9 (69)	29 (88)	46 (78)	
Preoperative symptoms	Hydrocephalic symptoms	8 (62)	10 (77)	19 (58)	37 (63)	0.333
	Visual symptoms	4 (31)	2 (15)	4 (12)	10 (17)	
	Cognitive dysfunction	4 (31)	1 (8)	3 (9)	8 (14)	
	Ataxia	1 (8)	2 (15)	2 (6)	7 (12)	
	Seizure	0 (0)	0 (0)	4 (12)	4 (7)	
	Incidental, no symptoms/deficits	3 (23)	0 (0)	6 (18)	9 (15)	
Hydrocephalus at initial diagnosis (MRI)	Yes	6 (46)	10 (77)	19 (58)	35 (59)	0.267
	No	7 (54)	3 (23)	14 (42)	24 (41)	
Tumor localization	Lateral ventricles	7 (54)	12 (92)	33 (100)	52 (88)	0.175
	Anterior horn	3 (23)	2 (15)	12 (36)	17 (29)	
	Posterior horn	1 (8)	1 (8)	5 (15)	7 (12)	
	Inferior horn	0 (0)	0 (0)	5 (15)	5 (8)	
	Body	0 (0)	5 (38)	5 (15)	10 (17)	
	Atrium	3 (23)	4 (31)	6 (18)	13 (22)	
	Third ventricle	6 (46)	6 (46)	8 (24)	20 (34)	
Therapy						
Tumor volume, median cm^3^	Preoperative	3.1 ± 2.8	20.1 ± 6.6	6.2 ± 5.0	9.5 ± 3.3	*0.001
	Postoperative	3.1 ± 2.8	2.3 ± 0.8	0.0 ± 0.0	0.0 ± 2.3	*0.001
Shunting	Permanent shunting	5 (38)	4 (31)	2 (6)	11 (19)	*0.018
	Transient EVD	0 (0)	3 (23)	5 (15)	8 (14)	0.211
	Internal shunting with initial biopsy	5 (38)	1 (8)	1 (3)	7 (12)	
	Postoperative shunting	0 (0)	4 (31)	1 (3)	5 (8)	
	Internal shunt	0 (0)	1 (8)	0 (0)	1 (2)	
	Ventriculoperitoneal shunt	0 (0)	3 (23)	1 (3)	4 (7)	
Adjuvant therapy	Watch-and-wait	6 (46)	8 (62)	25 (76)	39 (66)	*0.049
	Radiotherapy	1 (8)	3 (23)	4 (12)	8 (14)	
	Radiochemotherapy	1 (8)	2 (15)	3 (9)	6 (10)	
	Chemotherapy	1 (8)	0 (0)	1 (3)	2 (3)	
	Brachytherapy	2 (15)	0 (0)	0 (0)	2 (3)	
	Best supportive care	2 (15)	0 (0)	0 (0)	2 (3)	

Characteristics are given for all patients with supratentorial intraventricular tumors (*n* = 59) as well as patients receiving biopsy (*n* = 13), incomplete (*n* = 13) and complete (*n* = 33) tumor resection. Of note, one patient undergoing incomplete tumor resection received both, internal shunting with biopsy, followed by VP shunting after resection. Significant differences are indicated by *p ≤ 0.05

Abbreviations: *EVD* external ventricular drainage, *MRI* magnetic resonance imaging, *KPS* Karnofsky Performance Status

Tumors were most commonly located in the lateral ventricles (52/59 patients, 88%), most frequently in the anterior horn (17/52 patients, 33%). Hydrocephalus due to tumors obstructing the foramina of Monro or the cerebral aqueduct was encountered in 35/59 patients (59%). Median preoperative tumor volume was 9.5 cm^3^ ± 3.3 (range: 0.2–101.0 cm^3^) in the entire patient cohort. Preoperative tumor volume was higher in patients presenting with hydrocephalus at the time of initial diagnosis (11.5 ± 5.0 versus 4.6 ± 2.7 cm^3^, *p* = 0.018), and tumor localization within the third ventricle was associated with an increased risk for preoperative hydrocephalus compared to tumors located within the lateral ventricles (*p* = 0.005). Mean ADC_mean_ value in the entire cohort was 1132 ± 63 × 10^−6^ mm^2^/s and did not differ significantly between patients treated with either stereotactic biopsy, complete, or incomplete tumor resection (982 ± 86 versus 1313 ± 156 versus 1120 ± 87 × 10^−6^ mm^2^/s, *p* = 0.209). Notably, higher tumor/CSF ratio in ADC_mean_ values on preoperative MRI was associated with less aggressive tumors histology (i.e., WHO CNS grade 1 or 2, epidermoid tumors, xanthogranuloma) compared to more aggressive tumors (i.e., WHO CNS grade 3 and 4, lymphoma, neuroendocrine metastasis; 0.382 ± 0.021 × 10^−6^ mm^2^/s versus 0.289 ± 0.034, *p* = 0.007).

### Surgical tumor resection and postoperative deficits

Patients either received microsurgical tumor removal (46/59 patients; 78%) or stereotactic biopsy only (13/59 patients; 22%) per recommendation of the interdisciplinary tumor board. Male-to-female ratio, preoperative KPS, and preoperative symptom burden did not differ between these two surgical groups, except that patients receiving biopsy were older (55.5 ± 7.1 versus 37.4 ± 3.3 years, *p* = 0.015) (Table 1). Median preoperative tumor volume was higher in patients undergoing microsurgical resection (11.1 ± 4.0 versus 3.1 ± 2.8 cm^3^, *p* = 0.004). No difference was seen regarding the anatomical tumor localization (*p* = 0.175).

Complete tumor resection was achieved in 33/46 microsurgical cases (72%). In 13/46 patients (28%), resection was incomplete with a median residual tumor volume of 2.3 ± 0.8 cm^3^ corresponding to a median EOR of 88% (range, 31–98%). Of note, median preoperative tumor volume was significantly higher in patients with incomplete tumor resection (20.1 ± 6.6 versus 6.2 ± 5.0 cm^3^, *p* = 0.041). Interestingly, in ependymal tumors, surgery almost always yielded complete resection (12/13 patients, 92%), and all tumors with complete resection were at least in part located within one or both lateral ventricles (33/33 patients, 100%).

Depending on tumor localization within the ventricular system, a transfrontal transcortical approach via the middle frontal gyrus was most frequently chosen in 19/46 patients (41%), followed by a transparietal transcortical approach in 17/46 patients (37%). In case of tumors within the third ventricle, an infratentorial supracerebellar approach was commonly applied (6/46 patients, 13%). Other approaches included the transtemporal transcortical (3/46 patients, 7%) and anterior interhemispheric transcallosal route (1/46 patients, 2%). There was no significant difference in surgical approaches between patients with complete or incomplete tumor resection.

**Fig. 1:**
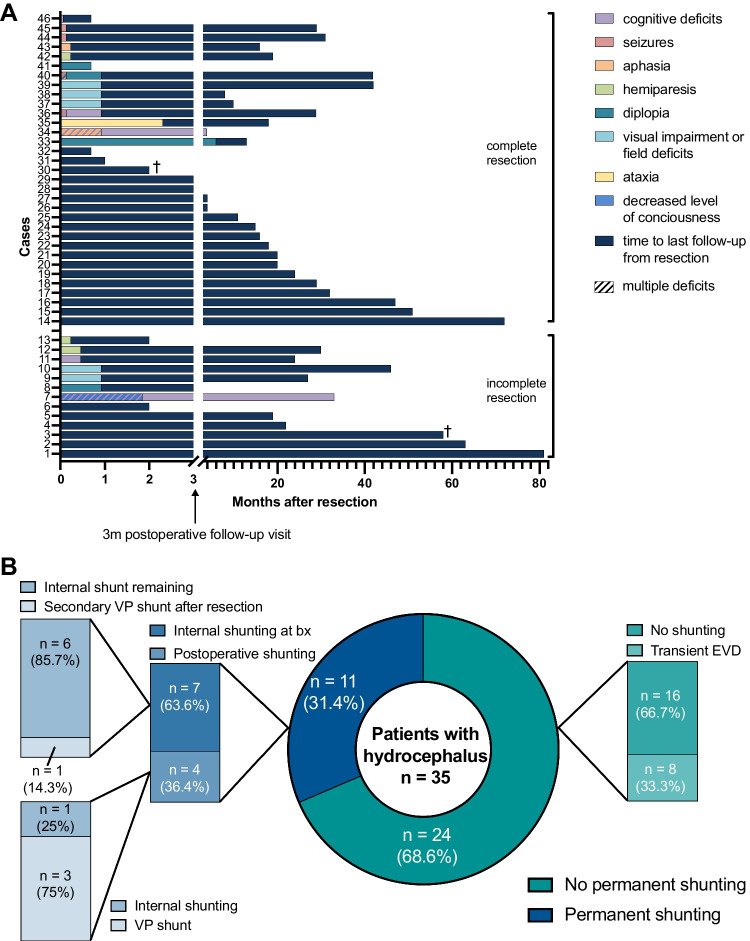
Perioperative morbidity and shunting strategies in patients with intraventricular tumors. **A** Postoperative follow-up of new neurological deficits after incomplete (*n* = 13) and complete (*n* = 33) tumor resection. Time intervals for different morbidities as well as time to last follow-up are color-coded according to the figure legend. **B** Distribution of shunting strategies in patients with supratentorial intraventricular tumors presenting with hydrocephalus per MRI (*n* = 35). Abbreviations: bx biopsy, EVD external ventricular drainage, VP ventriculoperitoneal

Postoperative follow-up demonstrated mostly transient mild to moderate new focal neurological deficits (Fig. 1A). Microsurgical tumor resection was associated with visual impairment or field deficits in 5/46 patients (11%), mild cognitive deficits in 5/46 patients (11%), and diplopia in 4/46 patients (9%). Severe neurological deficits were encountered in 4/46 patients (9%), including patients with status epilepticus, decreased level of consciousness, and aphasia necessitating prolonged ICU stays or additional surgery. Complete resection was not associated with an increased morbidity in our series (*p* = 0.484). No morbidity was seen in case of stereotactic surgery. New neurological deficits still present at follow-up visits 3 months after surgery were rare and only documented in 3/46 patients (7%). There was no significant difference in morbidity between different surgical approaches (*p* = 0.171).

### Neuropathological findings and adjuvant therapies

Overall, 27 different tumor types were diagnosed (Table 2) [16]. Ependymal tumors were diagnosed most frequently (13/59 patients; 22%), followed by tumors of glioneuronal and neuronal origin (12/59 patients, 20%).

**Table 2:** Histopathological diagnosis in patients with supratentorial intraventricular lesions

		Biopsy	Incomplete resection	Complete resection	Total	*p*-value
Overall, *n* (%)		13 (22)	13 (22)	33 (56)	59 (100)	
**Histology**	Ependymal tumors	0 (0)	1 (8)	12 (36)	13 (22)	0.068
	Subependymoma	0 (0)	0 (0)	8 (24)	8 (14)	
	Supratentorial ependymoma, YAP1 fusion-positive	0 (0)	0 (0)	2 (6)	2 (3)	
	Supratentorial ependymoma, NEC	0 (0)	1 (8)	2 (6)	3 (5)	
	Glioneuronal and neuronal tumors	3 (23)	3 (23)	6 (18)	12 (20)	
	Ganglioglioma	1 (8)	0 (0)	0 (0)	1 (2)	
	Central neurocytoma	1 (8)	2 (15)	3 (9)	6 (10)	
	Papillary glioneuronal tumor	1 (8)	0 (0)	2 (6)	3 (5)	
	Rosette-forming glioneuronal tumor	0 (0)	0 (0)	1 (3)	1 (2)	
	DNET	0 (0)	1 (8)	0 (0)	1 (2)	
	Gliomas	6 (46)	2 (15)	3 (9)	11 (19)	
	Pilocytic astrocytoma	2 (15)	1 (8)	2 (6)	5 (8)	
	Pleomorphic xanthoastrocytoma	0 (0)	1 (8)	0 (0)	1 (2)	
	Subependymal giant cell astrocytoma	1 (8)	0 (0)	0 (0)	1 (2)	
	Diffuse midline glioma, H3 K27-altered	1 (8)	0 (0)	0 (0)	1 (2)	
	Glioblastoma, IDH-wt	2 (15)	0 (0)	1 (3)	3 (5)	
	Pineal tumors	2 (15)	3 (23)	2 (6)	7 (12)	
	Pineocytoma	1 (8)	0 (0)	0 (0)	1 (2)	
	Pineoblastoma	0 (0)	1 (8)	1 (3)	2 (3)	
	Pineal parenchymal tumor of intermediate differentiation	0 (0)	2 (15)	1 (3)	3 (5)	
	Papillary tumor of the pineal region	1 (8)	0 (0)	0 (0)	1 (2)	
	Choroid plexus tumors	0 (0)	1 (8)	6 (18)	7 (12)	
	Choroid plexus papilloma	0 (0)	0 (0)	4 (12)	4 (7)	
	Atypical choroid plexus papilloma	0 (0)	0 (0)	1 (3)	1 (2)	
	Choroid plexus carcinoma	0 (0)	1 (8)	1 (3)	2 (3)	
	Atypical meningiomas	0 (0)	0 (0)	2 (6)	2 (3)	
	Epidermoid tumor	0 (0)	1 (8)	1 (3)	2 (3)	
	Germinoma	0 (0)	1 (8)	0 (0)	1 (2)	
	Adamantinomatous craniopharyngioma	0 (0)	1 (8)	0 (0)	1 (2)	
	Xanthogranuloma	0 (0)	0 (0)	1 (3)	1 (2)	
	Primary diffuse large B-cell lymphoma of the CNS	1 (8)	0 (0)	0 (0)	1 (2)	
	NET of unknown primary (metastasis to the brain)	1 (8)	0 (0)	0 (0)	1 (2)	

Histopathological tumor types according to the 2021 WHO classification of CNS tumors. Findings are given for all patients with supratentorial intraventricular tumors (*n* = 59) as well as patients receiving biopsy (*n* = 13), incomplete (≥ 1cm^3^ postoperative tumor volume; *n* = 13), and complete (< 1 cm^3^ postoperative tumor volume; *n* = 33) tumor resection

Abbreviations: *DNET* dysembryoplastic neuroepithelial tumor, *IDH-wt* isocitrate dehydrogenase wild type, *NET* neuroendocrine tumor

Most patients were initially followed via a watch-and-wait approach with surveillance scans according to the current standard-of-care given the rather benign nature of most tumors (39/59 patients, 66%). Adjuvant treatment included radiotherapy in 8/59 (14%) or radiochemotherapy in 6/59 patients (10%). Adjuvant treatment was selectively applied for tumors with a more aggressive histology (WHO grade III or IV, lymphoma, neuroendocrine metastasis). Tumors, in which complete resection was achieved, were associated with less aggressive therapy and more frequent utilization of watch-and-wait approaches postoperatively when compared to incompletely resected tumors and/or tumors that were only biopsied (*p* = 0.049). However, we cannot exclude bias due to small sample size and inequal distribution of histologies across resection categories, as benign tumors may be more amenable to more extensive resection.

### Resolution of preoperative hydrocephalus

**Fig. 2:**
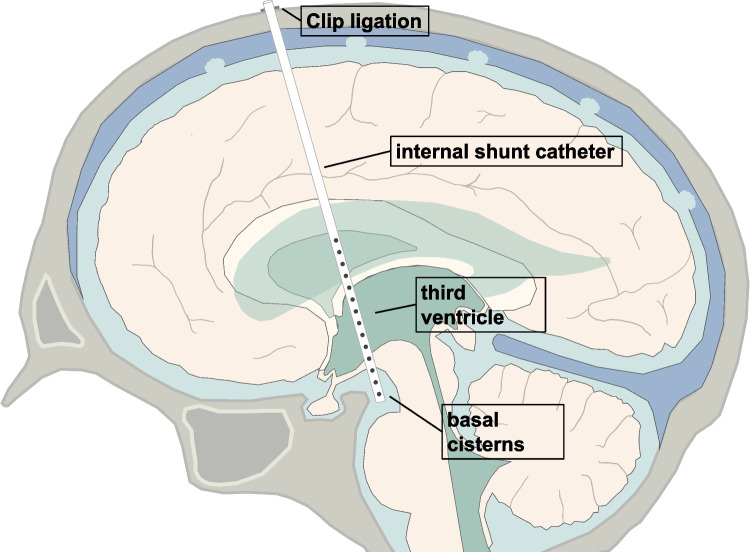
Stereotactic internal shunt placement. Schematic illustration demonstrating internal shunt implantation. After stereotactic trajectory planning, a ventricle catheter with multiple perforations is placed under stereotactic guidance to connect the supratentorial ventricular system with the basal cisterns and allow permanent CSF drainage. The catheter is subsequently closed and fixed extracranially using clip ligation

**Fig. 3:**
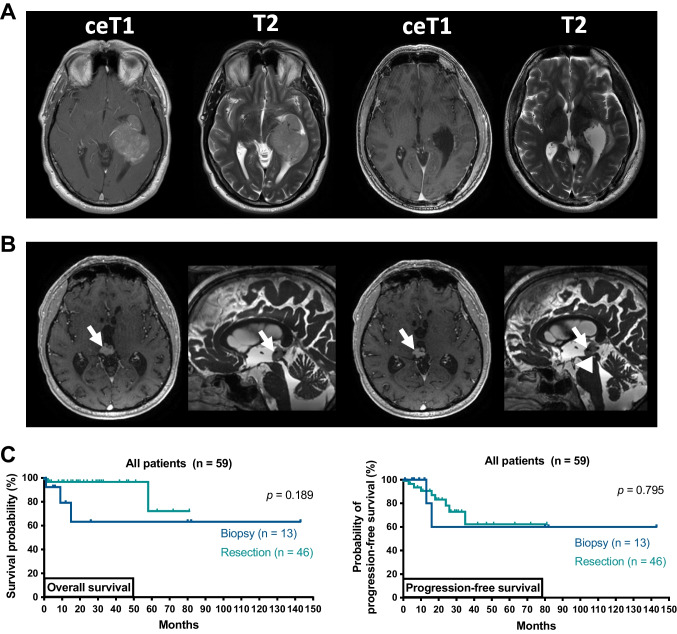
Outcome in patients depending on extent of resection. **A** Case example of a complete tumor resection: preoperative axial T1-weighted gadolinium enhanced and T2-weighted MRI showing an extensive contrast-enhancing (ce) lesion in the left inferior horn and atrium of the lateral ventricle consistent with an intraventricular meningioma (left). The patient underwent tumor resection via a parietal transcortical approach and complete resection was achieved (i.e., 100% of ce tumor volume, right). Histopathological examination confirmed meningioma and the patient was discharged without new focal neurologic deficits. **B** Case example of a stereotactic biopsy combined with internal shunt implantation: preoperative axial and sagittal MRI demonstrating a nodular lesion in the pineal region (arrows) with beginning obstructive hydrocephalus (left). Postoperative imaging demonstrating the internal shunt catheter (arrowhead) perforating the floor of the third ventricle and connecting the third ventricle with the basal cisterns (right). Histopathological findings demonstrated a papillary tumor of the pineal gland, and the patient was treated with radiotherapy. **C** Kaplan–Meier estimates of overall survival and radiographic progression-free survival in patients receiving biopsy (*n* = 13, dark blue) versus patients receiving tumor resection (*n* = 46, light blue). Tick marks indicate censored patients. Abbreviations: ceT1 T1-weighted gadolinium enhanced sequences, T2 T2-weighted sequences

Different peri- and postoperative shunting strategies were employed including the use of transient external ventricular drainage, permanent ventriculoperitoneal shunting, or stereotactic implantation of internal shunts, which was dependent on whether patients received biopsy or tumor removal (Fig. 1B, Table 1). Overall, 11/59 patients (19%) received any form of permanent shunting (i.e., VP or internal shunts). After microsurgical tumor resection, 5/46 patients (11%) needed additional permanent CSF drainage via VP shunts (4/46 patients, 9%, 3 of which had perioperative EVDs placed) or internal shunting (1/46 patients, 2%; Fig. 2). In all patients with incomplete tumor resection, residual tumor either impeded CSF flow through the foramina of Monro, or the cerebral aqueduct. Specifically, one patient received VP shunting after incomplete resection of an ependymoma and postoperative EVD-related ventriculitis with increased cell count and elevated protein levels. Furthermore, postoperative VP shunting was provided in two patients following intraoperative hemorrhage of a highly vascularized pineoblastoma in one case, and incomplete resection of a choroid plexus carcinoma due to brain stem infiltration in another case, both leading to obstructive hydrocephalus. Of note, two patients initially received biopsy along with internal shunting and subsequently underwent tumor resection. In one such case, additional secondary VP shunt implantation was performed for obstructive hydrocephalus due to postoperative bleeding and residual tumor impeding CSF flow after incomplete tumor resection of a pineal parenchymal tumor of intermediate differentiation (Table 1). Interestingly, postoperative follow-up imaging revealed that one patient exhibited an entrapped temporal horn caused by adhesions and scarring at the level of the atrium, and subsequently received internal shunting connecting the temporal horn with the basal cisterns. In addition, transient EVDs were placed intraoperatively in 8/46 patients (14%) undergoing tumor resection, but could be removed without any additional shunting thereafter. Complete tumor resection was associated with less permanent shunting in comparison to incomplete tumor resection, irrespective of tumor histology (6% versus 31%, *p* = 0.025; Fig. 3A).

In patients undergoing biopsy, internal shunting was used to alleviate hydrocephalic symptoms in 5/13 patients (38%; Fig. 3B). Resolution of hydrocephalic symptoms was observed in all patients, and 10/59 patients (17%) experienced a clinically relevant improvement in preoperative symptoms other than hydrocephalus.

### Long-term outcomes

Median follow-up was 18 months (range: 1–143 months). Overall, 10/59 patients (17%) suffered SIVT progression, and 5/59 (8%) patients died of tumor progression. Across the entire patient cohort, both median overall survival (OS) and median progression-free survival (PFS) were not reached (range: 1–143 months; Supplementary Fig. 2A). There was no difference between patients treated with resection or biopsy (OS: *p* = 0.189; PFS: *p* = 0.795; Fig. 3C). In particular, complete resection was not associated with improved OS or PFS (*p* = 0.399 and *p* = 0.237). These findings were also consistent when performing the analyses in patients ≥ 18 years of age (49/59 patients, 83%).

Moreover, we aimed to analyze the impact of the extent of resection on clinical outcomes depending on WHO grade. In tumors with lower grade histology (i.e., WHO CNS grade 1 or 2, epidermoid tumors, xanthogranuloma), comparison of OS was not possible due to missing death events. Furthermore, PFS did not differ between complete and incomplete tumor resection (*p* = 0.078). In tumors with higher grade histology (i.e., WHO CNS grades 3 and 4), complete resection was neither associated with improved OS nor PFS (*p* = 0.323 and *p* = 0.549, respectively; Supplementary Fig. 2B). No other statistically significant predictors of outcome were identified on univariate analysis.

## Discussion

Tumors of the ventricular system represent a surgical challenge due to their critical anatomic localization, clinical presentation with hydrocephalus, and the necessity of prompt surgical intervention. Accordingly, treatment decisions have to account for both establishing histological diagnosis and assessing feasibility of tumor resection, as well as evaluating acute and long-term shunting strategies. No “standard-of-care” exists, and optimal management of such tumors therefore remains unclear. In the current study, we elaborate on shunting strategies in patients presenting with hydrocephalus and report on surgical approaches and clinical outcomes.

Our analysis demonstrated that patients predominantly presented with symptoms and MRI findings consistent with hydrocephalus due to tumor mass effect on bottlenecks of CSF flow. Accordingly, preoperative tumor volume was associated with an increased risk for hydrocephalus due to the obstructive mass effect. Excitingly, permanent shunting could almost always be avoided when complete tumor resection was achieved, reducing the risk for postoperative VP shunt complications, secondary surgeries, and thus likely improving quality of life [11]. This observation therefore contradicts prior reports describing frequent persistent hydrocephalus, for example, due to scar tissue formation in choroid plexus tumors [2, 7], suggesting that surgical tumor resection should be combined with shunt placement. In contrast, close clinical and imaging follow-up is advised in cases of incomplete tumor resection with residual tumor impeding CSF flow, as almost every third patient required permanent shunt implantation to ameliorate persistent or recurrent hydrocephalus. As was the case in our patient cohort, this might not only be the result of residual tumor formation, but is also caused by tumor-related secretory proteins, tumor cells, intraventricular hemorrhage during surgery, or postoperative infections impeding CSF resorption [2]. When in doubt, perioperative EVD placement may represent a strategy to immediately alleviate hydrocephalic symptoms, while still providing time to re-evaluate the need for permanent shunting during the postoperative recovery period. On a cautionary note, we did not include infratentorial tumors located within the fourth ventricle or cerebellar aqueduct, which have been linked to higher postoperative shunt dependency [5, 8, 30].

We found that in cases in which microsurgical tumor resection appears feasible, complete tumor resection can frequently be achieved independent of tumor entity. It remains to be noted that 44% of patients had less than complete tumor resection. This might reflect the clinical paradigm that preservation of neurological function should be prioritized over extent of resection. The fimbria fornix in particular represents a critical structure at risk during surgery for SIVTs, and damage may clinically translate into short-term memory impairment including both verbal and visuospatial memory [29]. Accordingly, we encountered only 5/59 patients with new postoperative memory impairments or other cognitive deficits. Importantly, new postoperative neurocognitive impairments were assessed through subjective patient reports during follow-up visits, and more nuanced neuropsychological test batteries were not routinely utilized, thus limiting our findings. Overall, new postoperative neurological deficits were generally rare, transient, and mild in nature. This is in line with prior studies [1, 12, 18, 31] reporting that surgery on supratentorial intraventricular tumors might be considered safe and often yields sufficient results in regards to postoperative tumor volumes. Surgery therefore demonstrated a positive risk–benefit ratio enabling further adjuvant therapies whenever necessary. Concomitantly, we encountered excellent long-term survival. Considering continuous advances in minimally invasive neuroendoscopic approaches, both microsurgical resection or neuroendoscopy should be carefully evaluated in these cases [24]. Interestingly, lower normalized ADC_mean_ values determined on preoperative MRI were associated with higher WHO grades and might therefore be utilized for preoperative prognostication and working diagnosis. When lesions appeared not surgically approachable or patients were not surgical candidates for other reasons (e.g., low clinical performance score), biopsy enables tissue-based diagnosis to guide further medical therapies.

Neuropathological findings in our study cohort were diverse and consistent with the literature, depicting the real-life clinical landscape of supratentorial intraventricular tumors [25]. In this context, clinical and demographic findings in combination with preoperative imaging may guide differential diagnosis and initial treatment evaluation. Collectively, tissue sampling for histopathological diagnosis and molecular characterization of SIVTs is almost always necessary for further adjuvant treatment decisions. Complete tumor resection effectively decreases the need for shunting in patients presenting with hydrocephalus. In contrast to resolution of hydrocephalus, extent of resection seems to be less important from a neurooncological standpoint. Here, similar outcomes between patients with complete and incomplete tumor resections in tumors with lower grade histology might reflect the benign nature of such lesions, while adjuvant therapy seems to play a crucial role in more aggressive tumors. Of note, one has to account for possible bias in these analyses due to small sample size and imbalances in tumor histology and adjuvant treatment regimens between patients receiving complete or incomplete tumor resection. In selected cases of pineal masses suspicious for central nervous system germ cell tumors such as germinoma, diagnostic workup should be complemented by CSF studies and biopsy, avoiding radical surgery [9]. Overall, biopsy (in combination with internal shunting when necessary) seems to be a valuable tool for tissue-based confirmation of diagnosis in medically treatable tumor entities.

Key limitations of this study included the small sample size and the inclusion of patients under the age of 18 in our cohort due to similar perioperative hydrocephalus and morbidity management, respectively [11]. For these reasons, the conclusions regarding clinical outcomes and effects of neurooncological treatment regimens may be limited in scope. Although we were able to extract information on the specific postoperative deficit, the retrospective nature of our study did not allow to calculate more advanced neurological assessment scales such as MDASI-BT, EORTC QLQ-BN20, or NANO. As a result, we were not able to provide an additional early endpoint of symptom resolution prior to the 3-month follow-up visit. Moreover, we did not include endoscopic surgery for resection of intraventricular tumors or third ventriculostomy, which can be promising in selected cases [26]. In this context, neuroendoscopic procedures represent a promising and evolving field, providing a minimally invasive approach for tumor resection while also presenting an opportunity to treat obstructive hydrocephalus via third ventriculostomy and thus reducing the need for permanent shunting [24, 32]. Randomized prospective studies to evaluate surgical outcome and shunting strategies remain difficult to conduct given the rarity of supratentorial intraventricular lesions. As a next step, retrospective multicenter studies including patients with intraventricular tumors should be conducted to evaluate shunting strategies in a larger patient cohort.

## Conclusion

In conclusion, our data show that microsurgical tumor resection can be safely performed in patients presenting with hydrocephalus, deferring the need for primary shunt placement if complete resection is possible. In patients where surgical resection is not deemed feasible, stereotactic biopsy in combination with internal shunting represents an effective approach to establish diagnosis and ameliorate hydrocephalus-related symptoms. Clinical outcomes after microsurgical tumor resection were excellent when appropriate adjuvant therapy was provided, and new postoperative focal neurological deficits were characteristically mild and transient in nature.

## Supplementary Information

Supplementary file1: Supplementary figure 1. Flow diagram of patient selection. Schematic representation of the formation of a selected patient cohort exclusively including patients with supratentorial intraventricular tumors receiving biopsy or microsurgical tumor resection treated at the Center for Neuro-Oncology at the Ludwig-Maximilians-University School of Medicine between 2014 and 2022 (*n* = 59).

Supplementary file2: Supplementary figure 2. Outcome depending on extent of resection and histology. A-B: Kaplan-Meier estimates of overall survival and radiographic progression-free survival in the entire cohort (*n* = 59; A) and in patients receiving tumor resection with high grade tumor (WHO grade 3 and 4, lymphoma, neuroendocrine metastasis, *n* = 16) with curves displayed for patients with incomplete tumor resection (*n* = 10, blue) and complete tumor resection (*n* = 6, red; B). Note the different range on the x-axis in B. Tick marks indicate censored patients.

## Abbreviations

ADC: Apparent diffusion coefficient
ANOVA: One-way analysis of variance
CE: Contrast enhancing
CNS: Central nervous system
CpG: Cytosine-guanine dinucleotide
CSF: Cerebrospinal fluid
EOR: Extent of resection
EVD: External ventricular drainage
KPS: Karnofsky performance status
MGMT: O6-Methylguanine-DNA-methlytransferase
MRI: Magnetic resonance imaging
OS: Overall survival
PFS: Progression-free survival
PPTID: Pineal parenchymal tumors of intermediate differentiation
ROI: Region of interest
SIVT: Supratentorial intraventricular tumor
VP shunt: Ventriculoperitoneal shunt
WHO: World Health Organization
