# Supplementary figures and images for: Shunt dependency in supratentorial intraventricular tumors depends on the extent of tumor resection
Source: Acta Neurochir (Wien). 2023 Mar 2;165(4):1053–64. doi: 10.1007/s00701-023-05532-7 (PMC10068640; doi:10.1007/s00701-023-05532-7)

## Supplementary figure 1

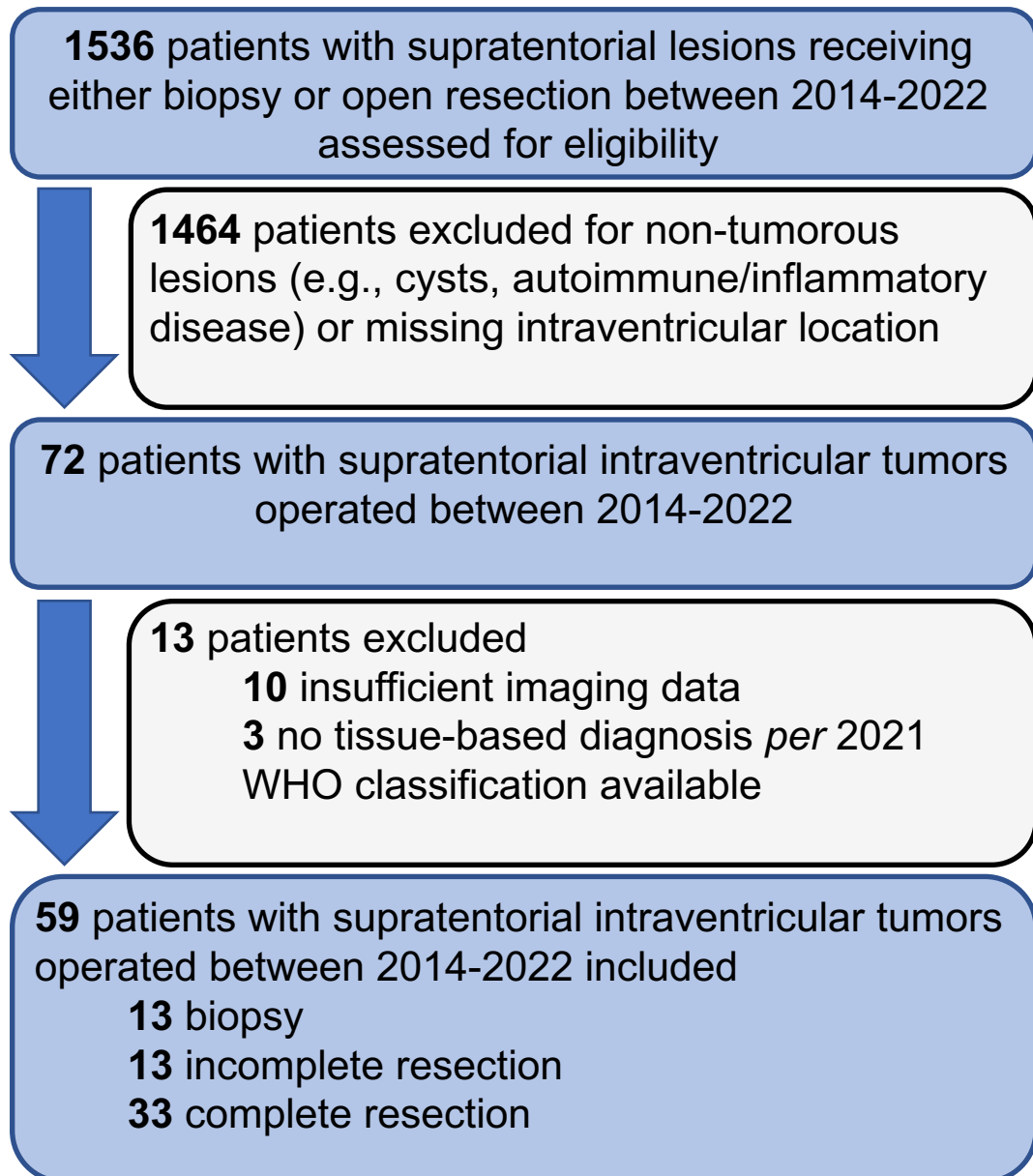

Supplement: Supplementary file 1 — Supplementary file1: Supplementary figure 1. Flow diagram of patient selection. Schematic representation of the formation of a selected patient cohort exclusively including patients with supratentorial intraventricular tumors receiving biopsy or microsurgical tumor resection treated at the Center for Neuro-Oncology at the Ludwig-Maximilians-University School of Medicine between 2014 and 2022 (n = 59). [file 701_2023_5532_MOESM1_ESM.pdf]

# Supplementary figure 2

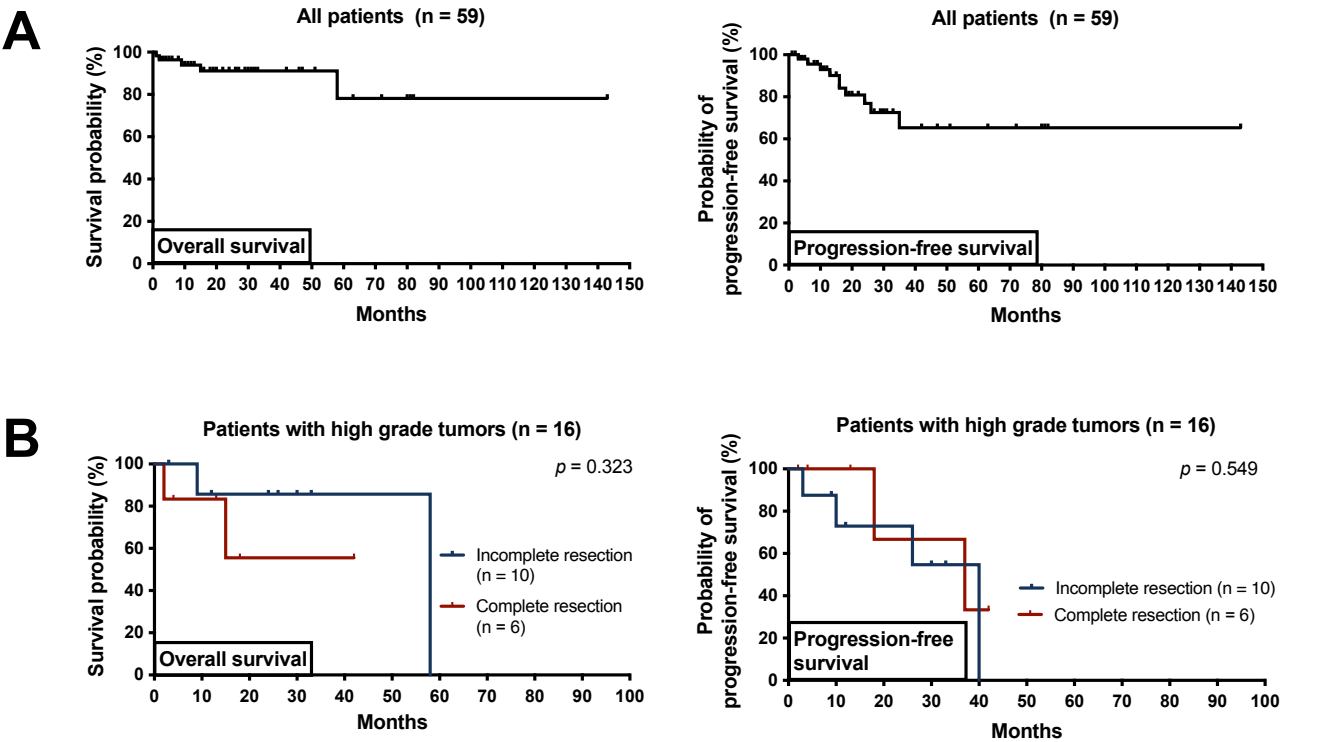

Supplement: Supplementary file 2 — Supplementary file2: Supplementary figure 2. Outcome depending on extent of resection and histology. A-B: Kaplan-Meier estimates of overall survival and radiographic progression-free survival in the entire cohort (n = 59; A) and in patients receiving tumor resection with high grade tumor (WHO grade 3 and 4, lymphoma, neuroendocrine metastasis, n = 16) with curves displayed for patients with incomplete tumor resection (n = 10, blue) and complete tumor resection (n = 6, red; B). Note the different range on the x-axis in B. Tick marks indicate censored patients. [file 701_2023_5532_MOESM2_ESM.pdf]
